# Supplementary material for: Graphene as Nanocarrier for Gold(I)-Monocarbene Complexes: Strength and Nature of Physisorption
Source: Molecules. 2023 May 7;28(9):3941. doi: 10.3390/molecules28093941 (PMC10180098; doi:10.3390/molecules28093941)
Supplement: Supplementary file 1 [file molecules-28-03941-s001.zip › molecules-2373121-supplementary.pdf]

Supplementary Material

Graphene as Nanocarrier for Gold(I)-Monocarbene Complexes: Strength and Nature of Physisorption

Cahit Orek[1], Massimiliano Bartolomei[2], Cecilia Coletti[3] and Niyazi Bulut[1]

1 Department of Physics, Faculty of Science, Firat University, Elazig 23119, Turkey

2 Instituto de Fisica Fundamental, Consejo Superior de Investigaciones Cientificas (IFF-CSIC), Serrano 123, 28006 Madrid, Spain

3 Dipartimento di Farmacia, Università degli Studi "G. d'Annunzio" Chieti-Pescara, Via dei Vestini, 66100 Chieti, Italy

Table. S1 Interaction energies between IMeAuCl - $C_{37}H_{16}$ as a function of the distance R between their centers of mass. All values are corrected for the basis set superposition error (BSSE).

R (Å)	Energy (meV)				
	**MP2C/CBS**	**DFT-SAPT/CBS**	**PBE-D3(BJ)/6-311++G(2p,2d)**	**M062X/6-311++G(2p,2d)**	**B3LYP-D3(BJ) /6-311++G(2p,2d)**
2.5	2875	2591	2969	3288	3075
2.75	507	626	734	735	572
3.0	−326	−250	−257	−343	−503
3.25	−703	−616	−622	−670	−856
3.5	−774	−679	−694	−656	−879
3.75	−676	−615	−643	−521	−772
4.0	−554	−512	−550	−376	−632
4.25	−440	−410	−453	−262	−501
4.5	−346	−329	−366	−182	−392
4.75	−271	−260	−292	−128	−306
5.0	−214	−205	−232	−93	−241

5.25	−169	−162	−183	−68	−190
5.5	−135	−131	−145	−49	−151
5.75	−109	−104	−114	−35	−121
6.0	−88	−84	−91	−24	−98
6.25	−72	−67	−72	−17	−78
6.5	−59	−57	−58	−11	−64
6.75	−49	−45	−46	−7	−52
7.0	−41	−39	−37	−5	−42
7.25	−33	−31	−30	−3	−34
7.5	−28	−26	−24	−1	−28
7.75	−23		−20	0	−23
8.0	−20		−16	1	−18

Table S2. Physical contributions to the total interaction energy as a function of the distance R between the centers of mass of IMeAuCl and $C_{37}H_{16}$ calculated at the DFT−SAPT/CBS level of theory. All values are in meV.

R	Electr.	Exch.	Ind.	Disp. (Ed*f)[1]	δHF	Total
2.50	−6333.19	14126.86	−1388.80	−4628.11	65.12	2590.93
2.75	−3101.15	7004.94	−620.94	−3226.49	47.29	625.85
3.00	−1511.49	3447.02	−312.58	−2282.59	40.08	−250.29
3.25	−733.29	1686.04	−184.36	−1633.13	−15.52	−616.06
3.50	−353.58	820.17	−126.37	−1181.10	−29.71	−679.43
3.75	−168.74	398.35	−95.64	−862.88	−25.96	−615.28
4.00	−79.18	192.33	−76.04	−637.95	−14.11	−511.74

4.25	−35.46	93.32	−61.79	−476.54	−7.10	−410.48
4.50	−14.26	44.59	−50.83	−360.42	−6.20	−328.81
4.75	−3.79	22.06	−41.94	−275.86	−4.81	−259.71
5.00	1.40	10.81	−34.74	−213.69	−3.74	−205.39
5.25	3.84	5.52	−28.98	−167.24	−2.35	−162.15
5.50	5.07	2.51	−24.31	−132.34	−3.06	−130.72
5.75	5.65	1.05	−20.51	−105.71	−1.86	−104.28
6.00	5.95	0.62	−17.36	−85.30	−2.10	−84.39
6.25	6.05	1.45	−14.72	−69.42	−1.84	−67.25
6.50	6.02	1.13	−12.56	−56.93	−3.68	−56.81
6.75	5.93	0.05	−10.77	−47.03	−0.96	−45.17
7.00	5.82	0.22	−9.26	−39.13	−2.5	−38.52
7.25	5.68	0.33	−8.01	−32.76	−1.57	−31.03
7.50	5.52	1.34	−6.93	−27.58	−2.54	−25.73

[1]E_d value was multiplied by a factor of 1.1931

Cartesian Coordinates of the structures in Figure 5

$C_{37}H_{16}$ – IMeAuCl

70

C	−1.39682	0.21224	−0.17447
C	−0.87902	−1.11286	−0.12381
C	0.51692	−1.32511	0.05713
C	1.39687	−0.21221	0.17443
C	0.88040	1.11298	0.11283
C	−0.51831	1.32506	−0.04618
C	−1.03673	2.65583	−0.10943
C	−2.43628	2.86659	−0.26919
C	−3.31651	1.75163	−0.39752
C	−2.80135	0.42522	−0.33460
C	−3.67940	−0.68913	−0.46102
C	−3.16069	−2.01681	−0.40962
C	−1.76389	−2.23062	−0.23092
C	−1.24322	−3.55575	−0.19070
C	0.15548	−3.76843	−0.00960
C	1.03819	−2.65574	0.09736
C	2.43544	−2.86666	0.27601
C	3.31692	−1.75156	0.39429
C	2.80136	−0.42516	0.33464
C	3.67900	0.68914	0.46429
C	3.16154	2.01686	0.40280
C	1.76247	2.23050	0.24302
C	1.24424	3.55573	0.18195
C	−0.15657	3.76833	0.01834
C	−0.68265	5.10206	−0.03884
C	−2.06555	5.28812	−0.20769
C	−2.95244	4.20419	−0.32626
C	−4.36730	4.38160	−0.49850
C	−5.21424	3.31024	−0.61189
C	−4.72644	1.96018	−0.56337
C	−5.57645	0.84675	−0.67858
C	−5.08729	−0.47005	−0.63045
C	4.04344	3.14187	0.52717
C	3.51052	4.44137	0.47373
C	2.13457	4.67422	0.30624
C	1.57836	5.99723	0.24916
C	0.23333	6.20149	0.08442
H	−2.46317	6.30670	−0.24956
H	−0.16820	7.21760	0.04344
H	−4.76068	5.40093	−0.53860
H	−6.28817	3.46885	−0.74191
H	−6.65075	1.00980	−0.80759
H	4.18646	5.29681	0.56688
H	2.25733	6.84924	0.34119
H	5.09482	2.99323	0.65911
H	4.18046	0.58869	1.40415

H	4.36641	−1.91162	0.52795
H	2.82416	−3.86251	0.32162
H	0.54195	−4.76466	0.04592
H	−1.90189	−4.39235	−0.29637
H	−3.82149	−2.85293	−0.50535
H	−5.75716	−1.29969	−0.71915
H	4.41678	0.60155	−0.30572
Au	−3.24098	1.65009	3.10974
Cl	−5.51910	1.99747	2.96653
N	−0.29876	2.29210	3.37633
N	−0.62055	0.15695	3.31833
C	−1.28207	1.35066	3.26662
C	0.94651	1.69792	3.50049
C	0.74343	0.35050	3.46388
C	−0.53726	3.72995	3.40499
C	−1.27250	−1.14609	3.27224
H	1.85871	2.27687	3.56382
H	−2.25844	−1.01934	2.81369
H	−0.67253	−1.83401	2.66662
H	1.44423	−0.47390	3.48860
H	−1.39370	−1.54783	4.28638
H	−0.54385	4.09482	4.44008
H	−1.51237	3.92455	2.94702
H	0.24454	4.24145	2.83299

$C_{54}H_{18}$ – IMeAuCl

89

C	−1.38630	0.21110	−0.16911
C	−0.87280	−1.10320	−0.11870
C	0.51300	−1.31340	0.04990
C	1.38516	−0.20952	0.16820
C	0.87192	1.10485	0.11630
C	−0.51402	1.31510	−0.05239
C	−1.02710	2.62980	−0.10420
C	−2.41350	2.84130	−0.27190
C	−3.28710	1.73570	−0.38710
C	−2.77240	0.42120	−0.33630
C	−3.64580	−0.68320	−0.45280
C	−3.13160	−1.99880	−0.40350
C	−1.74500	−2.20760	−0.23650
C	−1.23190	−3.52270	−0.18680
C	0.15540	−3.73280	−0.01860
C	1.02680	−2.62770	0.10030
C	2.41270	−2.83940	0.27020
C	3.28540	−1.73460	0.39030
C	2.77100	−0.42010	0.33840
C	3.64480	0.68300	0.45730
C	3.13160	1.99860	0.40380

C	1.74504	2.20827	0.23321
C	1.23300	3.52350	0.18019
C	−0.15450	3.73460	0.01150
C	−0.66740	5.04890	−0.04100
C	−2.04680	5.24250	−0.20840
C	−2.92650	4.15570	−0.32410
C	−4.30530	4.34800	−0.49020
C	−5.16870	3.25560	−0.60300
C	−4.67330	1.94510	−0.55330
C	−5.52730	0.83780	−0.66620
C	−5.03180	−0.47350	−0.61830
C	−5.88600	−1.57940	−0.73210
C	−5.37770	−2.87940	−0.68430
C	−4.00360	−3.10310	−0.52060
C	−3.47820	−4.40250	−0.47010
C	−2.10380	−4.62660	−0.30470
C	−1.57960	−5.92560	−0.25390
C	−0.20850	−6.13330	−0.08820
C	0.66910	−5.04680	0.03100
C	2.04770	−5.24020	0.19960
C	2.92600	−4.15380	0.32050
C	4.30350	−4.34800	0.49100
C	5.16590	−3.25610	0.61060
C	4.67050	−1.94560	0.56160
C	5.52460	−0.83960	0.67940
C	5.03000	0.47180	0.62960
C	5.88460	1.57650	0.74720
C	5.37790	2.87690	0.69300
C	4.00470	3.10170	0.52130
C	3.48080	4.40140	0.46470
C	2.10660	4.62660	0.29610
C	1.58340	5.92570	0.24170
C	0.21230	6.13430	0.07530
H	−2.44200	6.25500	−0.24860
H	−0.16620	7.15280	0.03690
H	−4.71720	5.35340	−0.53240
H	−6.23370	3.43510	−0.73070
H	−6.59490	0.99870	−0.79480
H	−6.95620	−1.43420	−0.85920
H	−6.06240	−3.71930	−0.77580
H	−4.14930	−5.25300	−0.56050
H	−2.23860	−6.78600	−0.34370
H	0.17250	−7.15110	−0.05230
H	2.44310	−6.25230	0.23860
H	4.71430	−5.35400	0.53180
H	6.23040	−3.43470	0.74220
H	6.59140	−1.00260	0.81090
H	6.95360	1.42930	0.88180
H	6.06280	3.71640	0.78590
H	4.15330	5.25120	0.55440
H	2.24240	6.78630	0.32930
Au	0.03903	0.63764	3.47540
Cl	1.43124	2.46427	3.72285

N	−2.51177	−0.93793	3.07682
N	−0.81190	−2.25689	3.26957
C	−1.15865	−0.93585	3.26256
C	−2.99712	−2.23174	2.97288
C	−1.92479	−3.06374	3.09460
C	−3.33882	0.26191	3.03813
C	0.54358	−2.75004	3.48075
H	−4.04100	−2.44560	2.78388
H	1.24170	−1.93519	3.26346
H	0.67470	−3.07038	4.52224
H	−1.85475	−4.14187	3.03255
H	0.73755	−3.59015	2.80490
H	−4.08145	0.17079	2.23779
H	−2.68750	1.11899	2.83881
H	−3.84391	0.40748	4.00155

$C_{96}H_{24}$ – IMeAuCl

137

C	−0.00810	−1.41020	0.00010
C	−1.23410	−0.71310	0.00060
C	−1.24220	0.69820	−0.00140
C	−0.02460	1.40950	−0.00140
C	1.19670	0.70780	−0.00050
C	1.20480	−0.69440	−0.00080
C	2.42450	−1.39070	−0.00150
C	2.46000	−2.79570	−0.00290
C	1.24790	−3.51160	−0.00170
C	0.02080	−2.82140	0.00030
C	−1.24020	−3.58850	0.00270
C	−2.46630	−2.89380	0.00480
C	−2.45660	−1.41800	0.00290
C	−3.67860	−0.72400	0.00280
C	−3.68680	0.68110	−0.00080
C	−2.47290	1.38900	−0.00280
C	−2.49980	2.86460	−0.00620
C	−1.28220	3.57330	−0.00490
C	−0.01210	2.82090	−0.00200
C	1.20670	3.52540	0.00070
C	2.42710	2.82370	0.00220
C	2.40800	1.41850	0.00070
C	3.70610	0.69880	0.00060
C	3.71410	−0.65570	−0.00110
C	5.00750	−1.38840	−0.00180
C	4.97830	−2.80320	−0.00410
C	3.75180	−3.51700	−0.00490
C	3.75070	−4.92840	−0.00770
C	2.47680	−5.67600	−0.00680
C	1.24840	−4.98710	−0.00300
C	0.02150	−5.69540	−0.00090

C	−1.21310	−4.99730	0.00220
C	−2.43520	−5.70330	0.00400
C	−3.66890	−5.00440	0.00730
C	−3.69030	−3.59370	0.00780
C	−4.92450	−2.89370	0.00980
C	−4.94580	−1.47810	0.00700
C	−6.16680	−0.77410	0.00780
C	−6.17530	0.70290	0.00210
C	−4.96240	1.42070	−0.00250
C	−4.95780	2.83630	−0.00790
C	−3.73180	3.55050	−0.00950
C	−3.72700	4.96120	−0.01320
C	−2.50160	5.67420	−0.01170
C	−1.27140	4.98230	−0.00680
C	−0.04520	5.69470	−0.00260
C	1.18990	5.00070	0.00200
C	2.41000	5.70400	0.00780
C	3.69260	4.97150	0.00970
C	3.71020	3.56030	0.00570
C	4.94500	2.86110	0.00520
C	4.99080	1.44680	0.00230
C	6.20860	0.74290	0.00180
C	6.21690	−0.66990	−0.00030
C	7.43340	−1.37730	−0.00090
C	7.40890	−2.78720	−0.00320
C	6.19930	−3.51070	−0.00530
C	6.17860	−4.91950	−0.00870
C	4.96900	−5.62800	−0.01050
C	4.94230	−7.10140	−0.01530
C	3.78200	−7.78040	−0.01460
C	2.48560	−7.08040	−0.00900
C	1.27260	−7.78470	−0.00630
C	0.03540	−7.10830	−0.00230
C	−1.18880	−7.80060	−0.00080
C	−2.41110	−7.11150	0.00170
C	−3.69120	−7.84230	0.00000
C	−4.85980	−7.17990	0.00410
C	−4.89020	−5.70620	0.00940
C	−6.10990	−5.01080	0.01400
C	−6.14550	−3.60440	0.01380
C	−7.36100	−2.88650	0.01640
C	−7.37800	−1.48330	0.01320
C	−8.64050	−0.72220	0.01510
C	−8.64820	0.62300	0.00910
C	−7.39450	1.39820	0.00110
C	−7.39400	2.80150	−0.00620
C	−6.18680	3.53300	−0.01050
C	−6.16790	4.93970	−0.01620
C	−4.95640	5.64890	−0.01710
C	−4.94310	7.12290	−0.02090
C	−3.78220	7.79860	−0.01940
C	−2.49400	7.08260	−0.01370
C	−1.27980	7.78580	−0.00910

C	−0.04780	7.10750	−0.00250
C	1.18110	7.79840	0.00500
C	2.40220	7.10840	0.01060
C	3.69020	7.82350	0.01960
C	4.85850	7.15830	0.02110
C	4.90260	5.68540	0.01410
C	6.12040	4.99140	0.01260
C	6.15760	3.58310	0.00800
C	7.37570	2.87420	0.00640
C	7.41660	1.46480	0.00340
C	8.62450	0.75070	0.00260
C	8.63290	−0.64880	0.00060
H	8.35350	−3.33010	−0.00340
H	9.58920	−1.16650	0.00020
H	7.11730	−5.47030	−0.01020
H	5.88930	−7.62980	−0.02030
H	3.77850	−8.86470	−0.01900
H	1.28980	−8.87310	−0.00770
H	−1.19090	−8.88910	−0.00200
H	−3.66850	−8.92630	−0.00640
H	−5.80160	−7.71740	0.00100
H	−7.04250	−5.57220	0.01660
H	−8.30480	−3.42930	0.01990
H	−9.57220	−1.27770	0.02220
H	−9.58600	1.16800	0.01150
H	−8.34390	3.33340	−0.00760
H	−7.10690	5.49020	−0.01870
H	−5.89110	7.64940	−0.02450
H	−3.77190	8.88290	−0.02200
H	−1.29450	8.87420	−0.01010
H	1.18560	8.88690	0.00640
H	3.67370	8.90770	0.02610
H	5.79900	7.69790	0.02850
H	7.05250	5.55330	0.01520
H	8.31370	3.42830	0.00800
H	9.57470	1.27990	0.00360
Au	−1.41810	−0.97730	3.44380
Cl	−3.41810	−2.14870	3.52310
N	0.47750	1.38230	3.32820
N	1.56920	−0.49120	3.34930
C	0.29870	0.02340	3.37250
C	1.82810	1.70620	3.28180
C	2.51740	0.52330	3.29420
C	−0.60820	2.35800	3.38830
C	1.88300	−1.91560	3.43320
H	2.17700	2.73240	3.20760
H	0.98280	−2.48170	3.15680
H	2.69690	−2.15490	2.73360
H	3.58260	0.32000	3.23140
H	2.17940	−2.18070	4.46070
H	−0.71810	2.74410	4.41430
H	−1.53840	1.85630	3.08760
H	−0.39600	3.18640	2.69700

$C_{150}H_{30}$ – IMeAuCl

197

C	0.81110	0.73810	−0.61830
C	1.98010	1.54810	−0.66480
C	−0.45860	1.33770	−0.57300
C	0.92780	−0.66220	−0.60970
C	1.86400	2.94830	−0.66660
C	−0.57620	2.75580	−0.57270
C	3.24990	0.94780	−0.70310
C	−1.61220	0.53800	−0.51800
C	2.21390	−1.26950	−0.64820
C	−0.22620	−1.46200	−0.55730
C	3.36780	−0.46960	−0.69600
C	0.57820	3.55590	−0.62000
C	−1.49590	−0.86210	−0.51090
C	3.01960	3.74860	−0.70640
C	−1.84570	3.35620	−0.51700
C	4.40470	1.74910	−0.74390
C	−2.89800	1.14560	−0.46170
C	2.32990	−2.66930	−0.63480
C	−0.10850	−2.88040	−0.54280
C	0.46160	4.95800	−0.61230
C	4.28770	3.14810	−0.74600
C	−3.01390	2.54600	−0.46150
C	4.63780	−1.06990	−0.73130
C	−2.66450	−1.67270	−0.44820
C	1.16190	−3.47960	−0.58240
C	2.89850	5.16950	−0.70200
C	−1.96070	4.75780	−0.51080
C	5.69100	1.13710	−0.78140
C	−4.05160	0.34530	−0.40110
C	3.60100	−3.26840	−0.67130
C	−1.26210	−3.68050	−0.48420
C	1.63510	5.76720	−0.65510
C	−0.80730	5.55670	−0.55880
C	5.80580	−0.25540	−0.77520
C	−3.93420	−1.07260	−0.39470
C	4.75260	−2.46820	−0.71970
C	−2.54790	−3.07330	−0.43620
C	5.46260	3.96310	−0.78650
C	−4.28450	3.14580	−0.40270
C	1.27730	−4.88110	−0.56730
C	4.06020	5.97420	−0.74200
C	−3.25020	5.36340	−0.45220
C	6.85130	1.94270	−0.82310
C	−5.32160	0.94660	−0.34320

C	3.71500	−4.68930	−0.65780
C	−1.14500	−5.08160	−0.46890
C	1.51830	7.17600	−0.64760
C	−0.92460	6.98260	−0.55060
C	5.34460	5.36230	−0.78560
C	−4.39820	4.56710	−0.39940
C	6.73050	3.36100	−0.82590
C	−5.43640	2.34550	−0.34450
C	7.08260	−0.85900	−0.81060
C	−5.08780	−1.87420	−0.33170
C	6.04490	−3.07870	−0.75530
C	−3.70280	−3.87340	−0.37360
C	2.56740	−5.48610	−0.60680
C	0.12420	−5.67970	−0.51180
C	0.23030	7.78040	−0.59600
C	7.19630	−2.27730	−0.80120
C	−4.97070	−3.27290	−0.32230
C	3.94140	7.37170	−0.73680
C	−3.36570	6.77250	−0.44580
C	8.11720	1.34170	−0.86010
C	−6.48980	0.13150	−0.28090
C	4.99270	−5.29210	−0.69500
C	−2.31750	−5.89040	−0.40800
C	2.67210	7.97200	−0.68960
C	−2.19430	7.58000	−0.49580
C	8.23250	−0.05830	−0.85400
C	−6.37420	−1.26190	−0.27560
C	6.15890	−4.47770	−0.74420
C	−3.58100	−5.29370	−0.36080
C	6.51030	6.16760	−0.82560
C	−5.67540	5.17030	−0.34010
C	7.89400	4.17010	−0.86670
C	−6.72860	2.95650	−0.28440
C	2.68380	−6.89460	−0.59240
C	0.24260	−7.10520	−0.49620
C	0.11180	9.19330	−0.58820
C	7.75820	5.55470	−0.86600
C	−6.84190	4.35620	−0.28340
C	8.47640	−2.88410	−0.83680
C	−6.14480	−4.08770	−0.25820
C	1.51290	−7.70240	−0.53740
C	5.13560	8.18100	−0.77870
C	−4.63380	7.36830	−0.38850
C	9.29430	2.17590	−0.90240
C	−7.76640	0.73570	−0.22300
C	5.10580	−6.68980	−0.68190
C	−2.19970	−7.29910	−0.39350
C	2.53840	9.40940	−0.68240
C	−2.30840	8.99300	−0.48860
C	6.35460	7.59100	−0.82130
C	−5.78750	6.56810	−0.33600
C	9.17150	3.52530	−0.90540
C	−7.88000	2.15480	−0.22540

C	9.53060	−0.68760	−0.88990
C	−7.53410	−2.06750	−0.21270
C	7.44090	−5.08100	−0.77990
C	−4.74160	−6.09830	−0.29850
C	3.95250	−7.49050	−0.63070
C	−0.91150	−7.90300	−0.43870
C	1.30910	9.97730	−0.63420
C	−1.15400	9.76790	−0.53550
C	9.63060	−2.03860	−0.88140
C	−7.41240	−3.48590	−0.20500
C	8.57100	−4.27180	−0.82570
C	−6.02620	−5.48690	−0.24770
C	4.96650	9.62940	−0.77240
C	−4.73730	8.80790	−0.38350
C	10.59080	1.50900	−0.93850
C	−8.91570	−0.06570	−0.16210
C	6.41660	−7.29230	−0.71950
C	−3.35270	−8.09510	−0.33340
C	1.62840	−9.11540	−0.52210
C	3.76410	10.19860	−0.72790
C	−3.61780	9.56980	−0.43190
C	−8.12340	4.95960	−0.22420
C	10.69970	0.18270	−0.93250
C	−8.79960	−1.46570	−0.15710
C	7.52190	−6.51030	−0.76610
C	−4.62240	−7.49540	−0.28590
C	−7.09760	7.17080	−0.27630
C	−9.15960	2.76210	−0.16670
C	4.05720	−8.93010	−0.61530
C	−0.79170	−9.31530	−0.42370
C	−8.20330	6.38930	−0.22380
C	−9.25320	4.15000	−0.16790
C	−8.57460	−4.29510	−0.14200
C	−7.19110	−6.29270	−0.18480
C	2.93820	−9.69240	−0.56370
C	0.47430	−9.89010	−0.46620
C	−6.07510	9.38550	−0.32470
C	−10.21300	0.56440	−0.10410
C	6.48830	−8.74860	−0.70340
C	−3.21800	−9.53190	−0.31980
C	−7.16800	8.62750	−0.27510
C	−10.31300	1.91560	−0.10730
C	−9.97550	−2.30030	−0.09410
C	−5.81570	−8.30510	−0.22270
C	5.39590	−9.50750	−0.65540
C	−1.98830	−10.09930	−0.36360
C	−8.43870	−5.67940	−0.13380
C	−9.85160	−3.64950	−0.08800
C	−7.03490	−7.71590	−0.17520
C	−11.38120	−0.30670	−0.04300
C	−4.44250	−10.32140	−0.25730
C	−11.27110	−1.63300	−0.03840
C	−5.64550	−9.75360	−0.21240

H	8.63980	6.16580	−0.89570
H	7.23970	8.19710	−0.85210
H	10.04950	4.14150	−0.93500
H	1.21490	11.04630	−0.62940
H	−1.24230	10.83730	−0.53050
H	10.59800	−2.50250	−0.90530
H	9.54110	−4.72970	−0.85010
H	5.85340	10.23150	−0.80520
H	11.46570	2.12850	−0.96780
H	3.66580	11.26650	−0.72440
H	−3.69990	10.63980	−0.42820
H	11.66450	−0.28520	−0.95660
H	8.49490	−6.96250	−0.79160
H	−9.17530	6.84210	−0.17990
H	−10.22250	4.60830	−0.12370
H	3.02100	−10.76230	−0.55170
H	0.56320	−10.95940	−0.45450
H	−6.15300	10.45520	−0.32300
H	7.46230	−9.19670	−0.73050
H	−8.14130	9.07620	−0.23270
H	−11.27980	2.37940	−0.06420
H	5.47450	−10.57700	−0.64350
H	−1.89320	−11.16820	−0.35300
H	−9.31950	−6.29060	−0.08650
H	−10.72890	−4.26570	−0.04130
H	−7.91920	−8.32210	−0.12830
H	−12.34570	0.16060	−0.00150
H	−4.34320	−11.38930	−0.24830
H	−12.14550	−2.25250	0.00690
H	−6.53160	−10.35600	−0.16670
Au	1.81810	−0.38990	2.89470
Cl	3.93710	−1.30690	2.81020
N	−0.34500	1.72360	2.92560
N	−1.20020	−0.25650	3.04960
C	−0.00600	0.40090	2.96240
C	−1.72010	1.88610	2.98840
C	−2.25930	0.63720	3.06670
C	0.61570	2.81920	2.87960
C	−1.33680	−1.70340	3.16470
H	−2.19180	2.85890	2.93970
H	−0.40340	−2.16290	2.82400
H	−1.51990	−1.98680	4.20900
H	−3.29130	0.31280	3.10000
H	−2.16640	−2.04510	2.53600
H	0.28510	3.56920	2.15250
H	1.58370	2.41040	2.57250
H	0.71450	3.27980	3.87080

Cartesian Coordinates of rigid $C_{37}H_{16}$ and IMeAuCl structures used in Figures 2 and 3

$C_{37}H_{16}$

53

C	0.00000	0.00000	0.00000
C	0.00000	0.00000	1.42360
C	1.23280	0.00000	−0.71180
C	1.23280	0.00000	2.13540
C	−1.23800	0.00000	2.13840
C	−1.23800	0.00000	−0.71480
C	2.46570	0.00000	0.00000
C	1.23280	0.00000	−2.14130
C	2.46560	0.00000	1.42350
C	1.23280	0.00000	3.56490
C	−1.23660	0.00000	3.56260
C	−2.47070	0.00000	1.42490
C	−1.23660	0.00000	−2.13910
C	−2.47070	0.00000	−0.00140
C	3.70370	0.00000	−0.71480
C	−0.00140	0.00000	−2.85220
C	2.46700	0.00000	−2.85220
C	3.70370	0.00000	2.13830
C	−0.00140	0.00000	4.27580
C	2.46700	0.00000	4.27580
C	−2.48180	0.00000	4.27550
C	−3.71070	0.00000	2.14690
C	−2.48180	0.00000	−2.85190
C	−3.71070	0.00000	−0.72340
C	4.93650	0.00000	−0.00140
C	3.70230	0.00000	−2.13910
C	0.00390	0.00000	−4.28710
C	2.46170	0.00000	−4.28710
C	4.93650	0.00000	1.42490
C	3.70230	0.00000	3.56260
H	−0.00170	−0.86310	4.90820
H	−0.00170	0.86310	4.90820
H	2.46760	0.00000	5.34580
C	−3.68710	0.00000	3.55230
H	−2.49280	0.00000	5.34540
C	−4.93570	0.00000	1.39700
C	−3.68710	0.00000	−2.12870
C	−2.44480	0.00000	−4.28770
C	−4.93570	0.00000	0.02660
H	5.86330	0.00000	−0.53580
H	4.62860	0.00000	−2.67450
C	1.23280	0.00000	−4.96930
C	−1.25800	0.00000	−4.97290

H	3.38280	0.00000	−4.83160
H	5.86330	0.00000	1.95940
H	4.62860	0.00000	4.09810
H	−4.63470	0.00000	4.09940
H	−5.88030	0.00000	1.94740
H	−4.63470	0.00000	−2.67590
H	−3.39390	0.00000	−4.83060
H	−5.88030	0.00000	−0.52390
H	1.23290	0.00000	−6.06350
H	−1.25360	0.00000	−6.06630

IMeAuCl

17

N	0.94150	3.52220	0.49390
C	−0.14680	3.49010	−0.33050
N	−1.21840	3.51660	0.51580
C	0.55640	3.57440	1.82360
C	−0.80670	3.57090	1.83740
C	2.32340	3.54500	0.03010
Au	−0.16700	3.45560	−2.31800
C	−2.60930	3.53190	0.07970
H	1.27450	3.57010	2.63330
H	−1.50820	3.56240	2.66150
H	2.93840	2.91620	0.68310
H	2.70820	4.57280	0.02860
H	2.34880	3.14810	−0.98990
Cl	−0.18180	3.31530	−4.62260
H	−3.20780	2.89900	0.74390
H	−2.99980	4.55760	0.08700
H	−2.65250	3.13620	−0.94000
